# Deciphering radiation effects in pap smears: A case report and review of challenges

**DOI:** 10.18632/oncoscience.636

**Published:** 2025-11-19

**Authors:** Gunvanti Rathod, Monica Mishra, Alisha Khan, Mishu Mangla

**Affiliations:** ^1^Department of Pathology and Lab Medicine, Additional Professor, AIIMS, Bibinagar, Telangana, India; ^2^Department of Pathology and Lab Medicine, Senior Resident, AIIMS, Bibinagar, Telangana, India; ^3^Department of Pathology and Lab Medicine, Junior Resident, AIIMS, Bibinagar, Telangana, India; ^4^Department of OBGY, Associate Professor, AIIMS, Bibinagar, Telangana, India

**Keywords:** papanicolaou smear, radiation cytology, cervical cancer, cytological changes, diagnostic pitfalls

## Abstract

Cervical carcinoma remains a major public health issue, especially in developing countries with limited access to screening. The Papanicolaou (Pap) smear is a cost effective, essential diagnostic tool for early detection and post-treatment surveillance of cervical lesions. Conization is used for early-stage disease, while advanced cases are managed with chemoradiation. In the report, a 44-year-old woman treated with hysterectomy and chemoradiation presented with a vault smear showing classic radiation-induced changes e.g. nuclear enlargement with preserved Nuclear: Cytoplasm ratio, cytoplasmic vacuolation and granularity, hyperchromasia with smudged chromatin, multinucleation, degenerative nuclear features including chromatin wrinkling, and occasional bizarre cells. Recognizing these features is vital to prevent misdiagnosis and unnecessary intervention. The present case highlights the need for heightened awareness of post-radiation cytology in clinical practice.

## INTRODUCTION

Cervical cancer ranks as the fourth most common malignancy among women worldwide, with a high burden in low-resource settings [1]. The primary etiological factor is persistent infection with high-risk Human Papilloma Virus (HPV), notably types 16 and 18 [2]. Early diagnosis using Pap smears and HPV testing has significantly improved patient outcomes. For advanced-stage disease, concurrent chemoradiation is the standard therapy. However, Radiation Therapy (RT) induces long-term cytological changes that can mimic High-Grade Squamous Intraepithelial Lesions (HSIL) or recurrence [3]. These radiation-induced atypia may persist for months to years, posing diagnostic challenges. It is imperative to distinguish benign post-radiation effects from malignant changes to avoid overtreatment [4]. These alterations can easily be mistaken for dysplastic or malignant cells, particularly by less experienced cytopathologists [5, 6]. Therefore, in post-radiation smears, it is essential to correlate cytological findings with the patient’s clinical history and treatment details, while maintaining a clear understanding of the potential diagnostic pitfalls. Such an approach helps to prevent unnecessary biopsies, misdiagnoses, and undue psychological distress for the patient. The present case report highlights the spectrum of cytological changes observed in a post-radiation smear from a patient previously treated for cervical carcinoma and emphasizes the clinical importance of accurately identifying these radiation-induced alterations.

## CASE REPORT

### Clinical presentation

A 44-year-old multiparous woman presented for routine follow-up one year after treatment for cervical carcinoma. She had been diagnosed with FIGO stage IV Squamous cell carcinoma of the cervix and underwent total abdominal hysterectomy with bilateral salpingo-oophorectomy. This was followed by external beam radiotherapy (EBRT) to 45 Gy in 25 fractions over five weeks, two sessions of intracavitary brachytherapy and concurrent weekly Cisplatin chemotherapy. The only symptom noted was mild urinary incontinence, which had gradually developed following treatment. A follow-up pelvic examination revealed no gross lesions or palpable masses. A vault smear was obtained for cytological evaluation.

### Cytological findings

**Figure 1:**
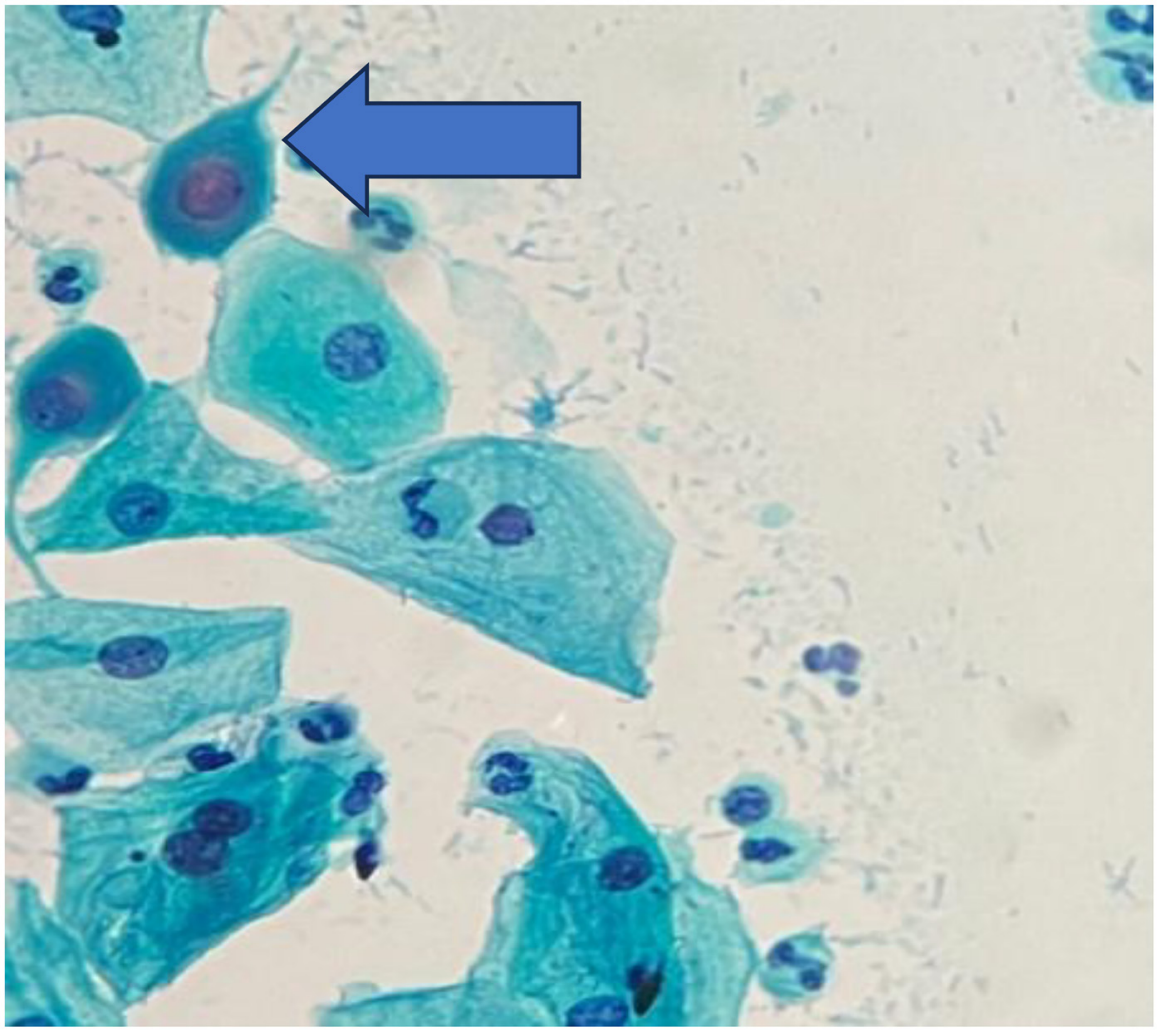
Pap smear shows cytoplasmic projection and nuclear smudging (Pap stain, 400×).

**Figure 2:**
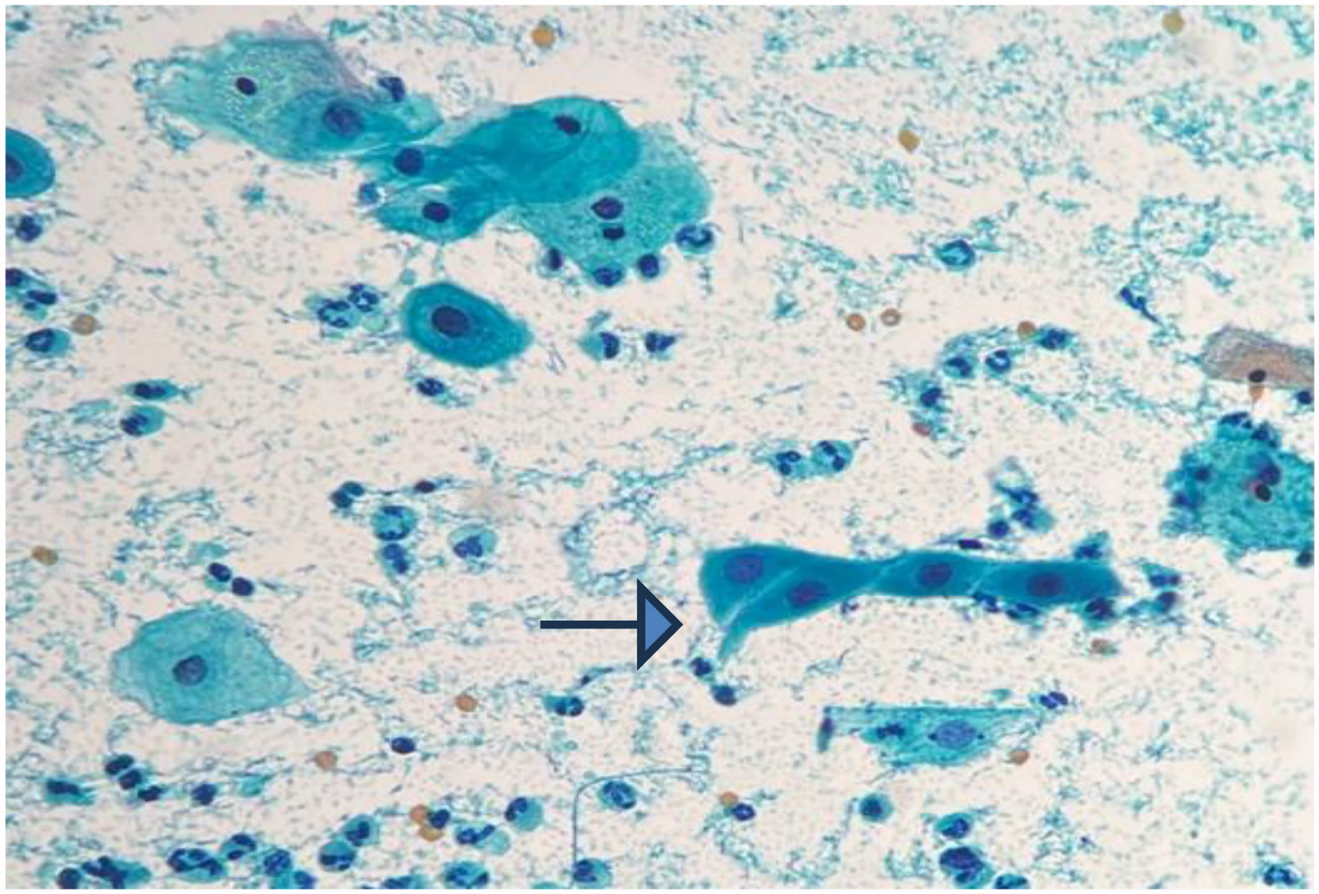
Smear shows cytoplasmic projection (Pap stain, 400×).

**Figure 3:**
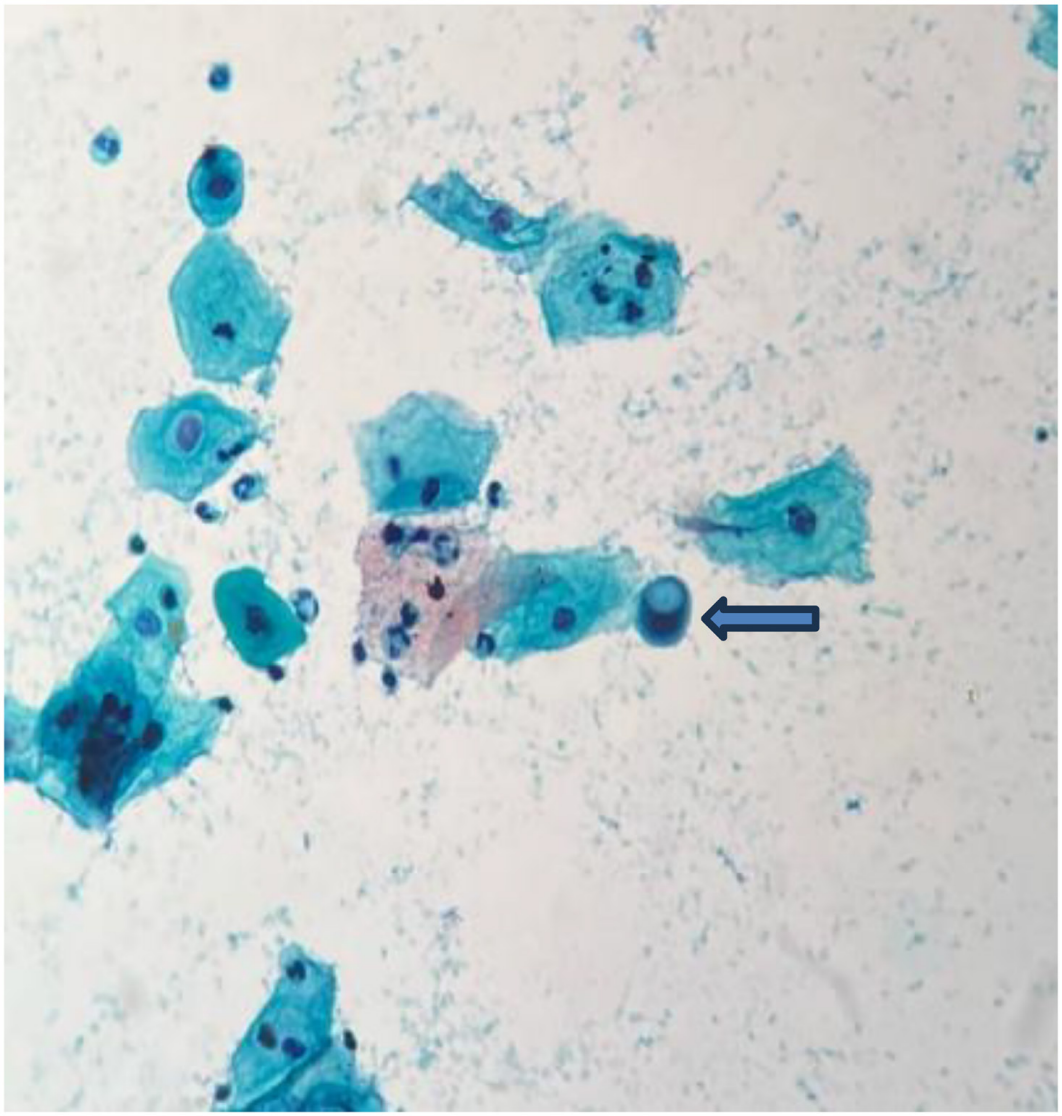
Smear shows a cell with cytoplasmic vacuolation (Pap stain, 400×).

The smear was prepared using the conventional Papanicolaou (Pap) staining method and examined microscopically. Microscopy revealed a moderately cellular smear. Numerous squamous epithelial cells exhibited: Nuclear enlargement with preserved N:C ratio, Cytoplasmic vacuolation and granularity, Mild hyperchromasia with smudged chromatin, Binucleation and multinucleation, Degenerative nuclear changes such as nuclear pallor, irregular membranes, and chromatin wrinkling, Occasional bizarre-shaped cells and Inflammatory background with atrophy. No malignant cells were seen. (Figures 1–3) Considering the patient’s history and the morphological features, final cytological diagnosis was given as Post-radiation changes, no evidence of malignancy. At 12-month follow-up, the patient remained clinically stable and asymptomatic. Continued cytological surveillance was advised.

## DISCUSSION

The Papanicolaou (Pap) smear remains an indispensable tool not only for the initial screening of cervical neoplasia but also for post-treatment surveillance. In women who have undergone radiation therapy for cervical cancer, cytological evaluation continues to play a crucial role in identifying persistent or recurrent disease. However, the utility of the Pap smear in this setting depends heavily on the cytopathologist’s ability to recognize and accurately interpret radiation-induced cellular alterations, which can mimic malignancy and pose significant diagnostic challenges [3]. Radiation therapy induces a wide spectrum of morphologic changes in epithelial cells, including nuclear enlargement, hyperchromasia, cytoplasmic vacuolation, and multinucleation [7]. These alterations may persist for several months to years following treatment and often overlap with features seen in high-grade squamous intraepithelial lesions (HSIL). Distinguishing between radiation-induced atypia and true dysplasia is critical, as misinterpretation may lead to unnecessary biopsies, over diagnosis, and psychological distress for the patient [8]. Accurate cytological interpretation in this context requires a thorough understanding of the patient’s treatment history, including the type, dose, and duration of radiation received. Additionally, knowledge of the temporal relationship between therapy and smear collection enhances diagnostic accuracy. Correlation with clinical findings, imaging studies, and, if necessary, histopathological confirmation remains essential in ambiguous cases. Incorporating these multidisciplinary inputs enables pathologists to offer a more nuanced and clinically relevant diagnosis, ultimately guiding appropriate patient management and avoiding overtreatment.

A mechanism of Radiation Damage is very important to know. Radiation therapy causes cellular injury via two major pathways: direct DNA double-strand breaks and indirect effects through the generation of reactive oxygen species (ROS). These ROS induce oxidative damage affecting cellular membranes, mitochondria, and nuclear content [9]. Characteristic radiation-induced cytological features include nuclear enlargement (nucleomegaly), multinucleation, hyperchromasia, smudged chromatin, cytoplasmic vacuolation, and eosinophilic cytoplasm [3]. These changes may persist up to 36 months post-therapy. Importantly, they usually maintain a preserved N: C ratio and lack mitotic activity [10, 11].

It’s a great diagnostic challenge to identify radiation-induced atypia. Misinterpretation of radiation-induced atypia as HSIL or recurrent carcinoma may result in false-positive reports, unnecessary biopsies, and patient anxiety [12]. Hence, correlation with clinical history, timing since therapy, and recognition of typical benign changes is essential for accurate diagnosis. Vault Pap smears remain a cornerstone in post-treatment surveillance, particularly after hysterectomy [13]. When atypical features are identified, especially in post-radiation settings, cautious interpretation is warranted. Literature emphasizes the need to differentiate reactive changes from recurrence to avoid over diagnosis.

## CONCLUSIONS

The present case illustrates the classical cytological hallmarks of radiation-induced changes in a post-treatment vault smear. It underscores the importance of recognizing benign post-therapeutic alterations and integrating cytological interpretation with the patient’s treatment history. This report contributes to existing literature by providing detailed cytomorphological descriptions from an Indian cohort, thereby enhancing awareness and diagnostic accuracy in radiation-related cytology.
